# Multi-biological functions of intermedin in diseases

**DOI:** 10.3389/fphys.2023.1233073

**Published:** 2023-09-06

**Authors:** Zhi Yang, Hongchun Li, Pengfei Wu, Qingyan Li, ChunYan Yu, Denian Wang, Weimin Li

**Affiliations:** ^1^ Precision Medicine Center, Precision Medicine Key Laboratory of Sichuan Province, State Key Laboratory of Respiratory Health and Multimorbidity, West China Hospital, Sichuan University, Chengdu, Sichuan, China; ^2^ Department of Nephrology, West China Hospital, Sichuan University, Chengdu, Sichuan, China; ^3^ National Chengdu Center for Safety Evaluation of Drugs, State Key Laboratory of Biotherapy/Collaborative Innovation Center for Biotherapy, West China Hospital, Sichuan University, Chengdu, China; ^4^ Institute of Respiratory Health, Frontiers Science Center for Disease-Related Molecular Network, West China Hospital, Sichuan University, Chengdu, Sichuan, China; ^5^ Frontiers Science Center for Disease-Related Molecular Network, West China Hospital, Sichuan University, Chengdu, Sichuan, China

**Keywords:** Intermedin (IMD), calcitonin gene-related peptide (CGRP), tumor, cardiovascular diseases, metabolic syndrome, sepsis

## Abstract

Intermedin (IMD) is a member of the calcitonin gene-related peptide (CGRP)/calcitonin (CT) superfamily, and it is expressed extensively throughout the body. The typical receptors for IMD are complexes composed of calcitonin receptor-like receptor (CLR) and receptor activity-modifying protein (RAMP), which leads to a biased activation towards Gα_s_. As a diagnostic and prognostic biomarker, IMD regulates the initiation and metastasis of multiple tumors. Additionally, IMD functions as a proangiogenic factor that can restrain excessive vascular budding and facilitate the expansion of blood vessel lumen, ultimately resulting in the fusion of blood vessels. IMD has protective roles in various diseases, including ischemia-reperfusion injury, metabolic disease, cardiovascular diseases and inflammatory diseases. This review systematically elucidates IMD’s expression, structure, related receptors and signal pathway, as well as its comprehensive functions in the context of acute kidney injury, obesity, diabetes, heart failure and sepsis. However, the precise formation process of IMD short peptides *in vivo* and their downstream signaling pathway have not been fully elucidated yet. Further in-depth studies are need to translate IMD research into clinical applications.

## 1 Introduction

In 2004, Roh and Takei first discovered intermedin (IMD), which is secreted and highly expressed in the intermediate lobe of the pituitary gland (Roh et al., 2004; Takei et al., 2004; Takahashi et al., 2006). IMD belongs to the calcitonin gene-related peptide (CGRP) superfamily, which includes members such as CGRP, calcitonin (CT), amylin (AMY), and adrenomedullin (ADM) (Chang et al., 2004; Bell et al., 2016). Mammalian IMD shares a sequence similar to fish ADM2, prompting some researchers to refer to it as ADM2.

**FIGURE 1:**
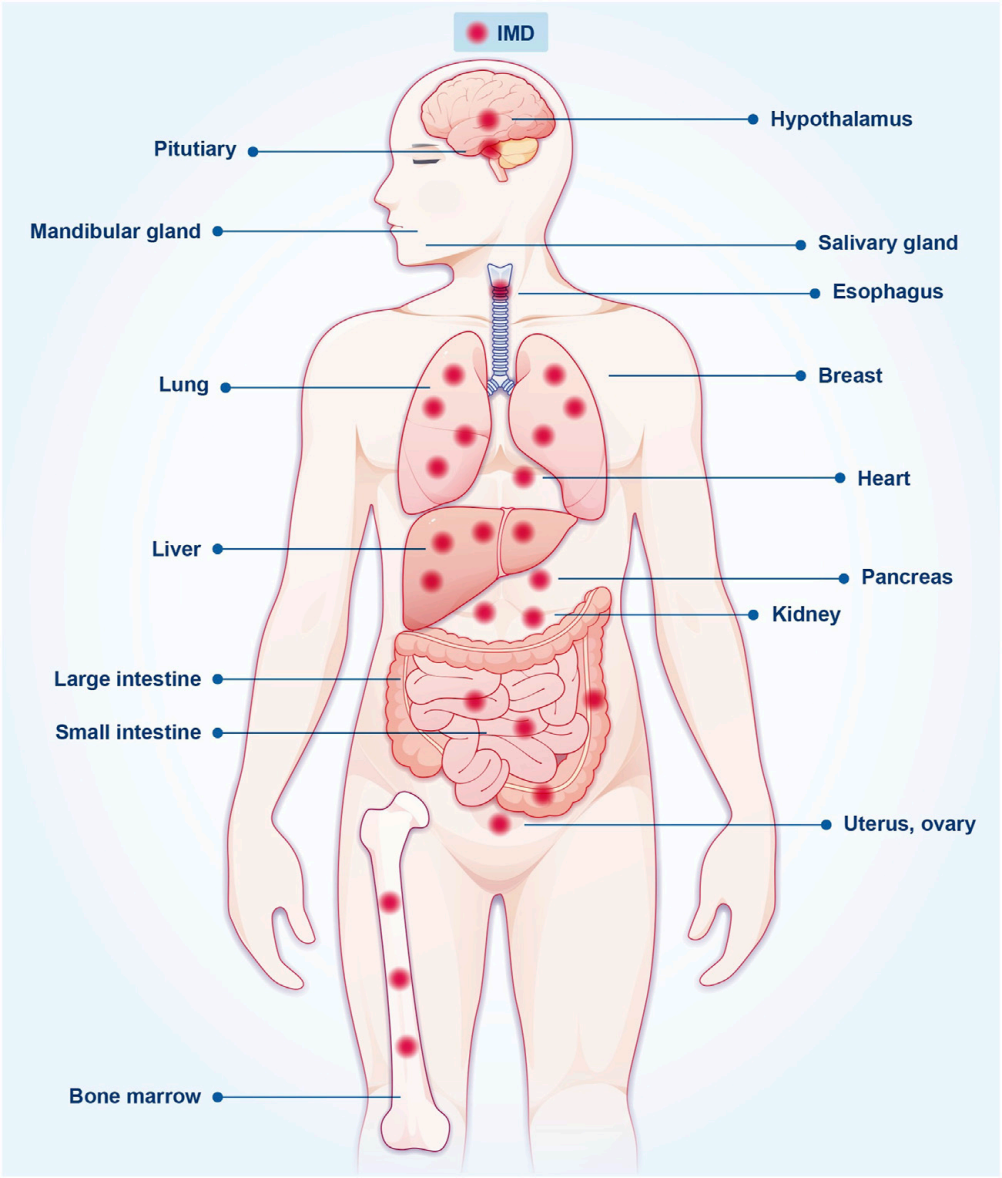
The expression of IMD is found in various organs and tissues throughout the body.

IMD is expressed in various tissues, such as the brain, kidney, liver, lung, submandibular gland, pancreas, esophagus, gastrointestinal tract, and adipose tissue (Figure 1) (Ogoshi et al., 2003; Ni et al., 2014). Also, IMD is expressed in multiple cell types, including cardiac fibroblasts, cardiomyocytes, cells of the cardiac microvasculature, arterial endothelial cells, renal mesangial cells, renal tubular epithelial cells, and others (Smith et al., 2009; Bell et al., 2012). However, its expression in plasma is relatively low, with levels ranging from 100 to 200 pg/mL (Hay et al., 2011; García-Ponce et al., 2016). Further studies are warranted to evaluate the underlying mechanisms of IMD peptides in the plasma and the enzymes responsible for their degradation.

The expression of IMD is regulated by an estrogen response element (ERE), a hypoxia response element (HRE), and an integrated stress response element (ISR) (García-Ponce et al., 2016). The expression of IMD increases in human aortic endothelial cells under various stress conditions, such as oxidative stress and serum starvation (García-Ponce et al., 2016). Studies have also found that in iodine-deficient rats, the expression of IMD was increased in thyroid follicular cells (Roh et al., 2004; Ni et al., 2014; Alexander et al., 2015).

Given its wide distribution, IMD may play critical biological roles. IMD mainly functions in an autocrine and paracrine manner, regulating hormone secretion and perfusion in major tissues and organs (Bell and McDermott, 2008). IMD is involved in regulating both prolactin secretion and estrogen-induced prolactin release (Roh et al., 2004). Additionally, IMD can regulate blood pressure in pregnant women (Alexander et al., 2015).

In this review, we systematically summarize the expression, molecular structure, receptors, signaling pathway of IMD, and its function in the context of the cardiovascular disease, metabolic syndrome, tumor blood vessel angiogenesis and remodeling, and inflammatory diseases. Our findings suggest that IMD may have broad clinical applications, but there are still some questions that need to be addressed in the future.

## 2 Formation of IMD

**FIGURE 2:**
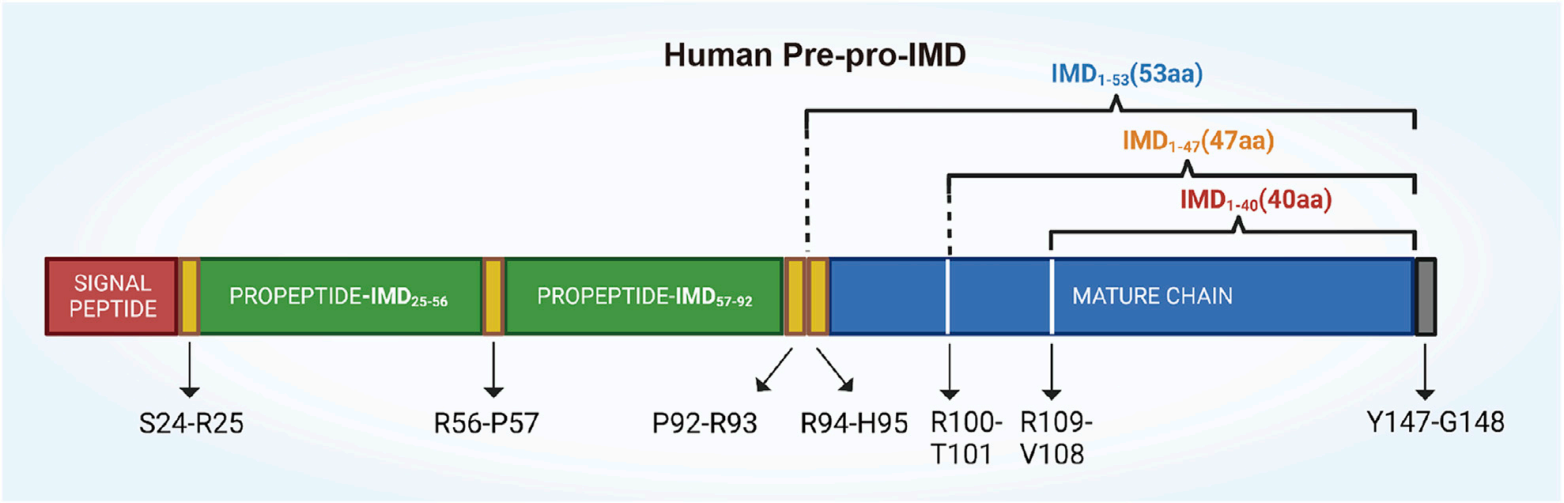
A schematic drawing illustrating the structure of the human IMD prepropeptide. The pre-pro-IMD molecule is cleaved at its C-terminal to yield three mature IMD peptides: IMD_1–53_, IMD_1–47_ (together referred to as IMD long), and IMD_1–40_ (known as IMD short). The precursor fragment, pre-pro-IMD_25–92_, is biologically inactive and undergoes cleavage at Arg_56_-Pro_57_, resulting in the formation of two fragments: propeptide-IMD_25–56_ and propeptide-IMD_57–92_.

The human IMD gene is located on chromosome 22q13.33 and it encodes a pre-pro hormone called pre-pro-IMD, which consists of 148 amino acids and a signal peptide for secretion at its N-terminus (Chang et al., 2004). Bell et al. discovered that *in vivo*, the complete pre-pro-hormone is hydrolyzed at three specific sites: Arg^100^-Thr^101^, Arg^107^-Val^108^, and Tyr^147^-Gly^148^. Notably, the site Tyr^147^-Gly^148^ undergoes amidation (Bell et al., 2016). The hydrolysis of the pre-pro-hormone produces three biologically active C-terminal peptides: IMD_1–40_, IMD_1–47_, and IMD_1–53_, along with several prerequisite fragments, such as IMD_25–56_ and IMD_57–92_ (Figure 2) (Ni et al., 2014).

As confirmed by sequence comparison, IMD is highly conserved across different species. The amino acid sequence of human IMD_1–47_ is 87% identical to that of rats and 60% similar to that of teleost fish (Ogoshi et al., 2003). While, compared to ADM of the CGRP family, only 28% of the sequences are identical. The properties of highly conserved to orthologs with low homology to paralogs indicate that IMD plays a unique role in the body (Ni et al., 2014; García-Ponce et al., 2016).

Studies have revealed that IMD and its related peptides exhibit unique synergistic and antagonistic effects on regulation of gene expression, biological processes, receptor binding, signal transduction, and the occurrence and development of diseases. Nevertheless, the precise formation process of these short peptides and their relative content *in vivo* have not been fully elucidated yet.

## 3 Receptors for IMD

IMD is a secretory peptide that is relatively short in length (Bell and McDermott, 2008; García-Ponce, Chánez Paredes et al., 2016). The typical receptors for peptides of CGRP family are complexes composed of calcitonin receptor-like receptor (CLR) and receptor activity-modifying protein (RAMP) (Roh et al., 2004). CLR belongs to the class B G protein-coupled receptor (GPCR) subunit, which enables IMD and other members of CGRP family to interact with cells and carry out their functions (Alexander et al., 2015). RAMPs function as molecular chaperones, facilitating the translocation of CLR from the endoplasmic reticulum and Golgi bodies to the cell surface (Weston et al., 2015). They enhance the cell surface expression of CLR and play a critical role in the pharmacology of CLR (Hay and Pioszak, 2016).

There are three types of RAMPs: RAMP1, RAMP2, and RAMP3. CLR interacts with different RAMPs, leading to varying ligand selectivity. RAMPs could alter CLR ligand selectivity through allosteric effects and direct peptide contacts (Booe et al., 2018). CLR heterodimerizes with RAMP1 to form the CGRP receptor, whereas CLR heterodimerizes with RAMP2 or RAMP3 to form the AM_1_ and AM_2_ receptors, respectively (Bell et al., 2016). The peptide ligands activate each receptor with differing potencies. CGRP has the highest affinity for the CGRP receptor, while ADM has the highest affinity for the AM_1_ and AM_2_ receptors. CGRP has also been observed to bind to AM_2_ receptor complexes, but with an affinity that is 50-fold lower than that of ADM(Hay et al., 2011). The human ADM was also able to stimulate cyclic adenosine monophosphate (cAMP) production via the CGRP receptor, but its affinity is approximately 10 times lower than that of CGRP (Bailey and Hay, 2006).

**FIGURE 3:**
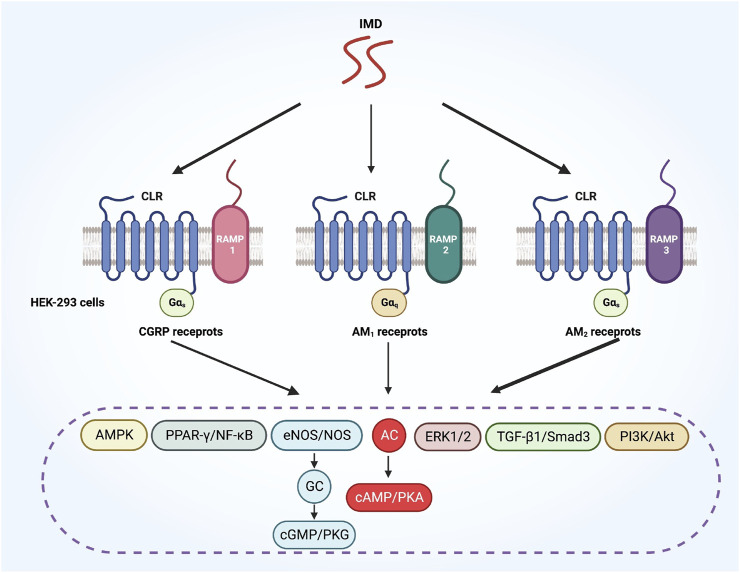
A diagram illustrates how IMD activates various downstream signaling pathways through different CLR/RAMP receptor complexes. CLR, calcitonin receptor-like receptor; RAMP, receptor activity-modifying protein.

IMD is capable of binding to all CGRP, AM_1_ and AM_2_ receptors (Zhang et al., 2016). Primarily, IMD signals via CGRP or AM_2_ receptors, and to a lesser extent, also via AM_1_ receptors (Figure 3) (García-Ponce et al., 2016; Roehrkasse et al., 2018). Although the AM_2_ receptor has the highest affinity for IMD, the majority of IMD’s cardiovascular effects are mediated by CGRP receptors and AM_1_ receptors, probably due to their high expression in the heart and blood vessels (Bell and McDermott, 2008). Furthermore, IMD may function through AMY receptors, which consist of the calcitonin receptor (CTR) and RAMPs (Owji et al., 2008). Since IMD interacts with multiple receptors, several regulatory mechanisms could be involved in mediating its biological effects. Therefore, designing specific inhibitors that target IMD based on its receptors is of great importance for its specific clinical applications (Zhang et al., 2018).

## 4 Signaling pathways for IMD

The CLR-based receptors are known for having complex downstream signaling pathways. The interaction of CLR with different G proteins initiates various signaling pathways (Alexander et al., 2015). RAMPs are involved in modifying the G protein selectivity activated by CLR-based receptors. For example, when the IMD_1-47_ polypeptide is added to HEK-293 cells, the CGRP and AM_2_ receptors lead to a biased activation toward Gα_s_ signaling, while the AM_1_ receptors show a bias toward Gα_q_ signaling (Figure 3) (Weston et al., 2016). Besides, CGRP, ADM and IMD may activate receptors via different signaling pathways depending on the specific peptide involved. For example, the AM_2_ receptors show a bias toward Gα_s_ signaling in response to ADM and change to Gα_q_ signaling in response to CGRP. Inversely, the CGRP receptors displays a bias toward Gα_s_ signaling when activated by CGRP, but changed to Gα_i_ signaling when activated by ADM (Weston et al., 2016).

It is likely that the main transduction pathway for CLR-based receptors activated by IMD involves coupling to Gs, activating adenylyl cyclase (AC), and resulting in intracellular accumulation of cAMP. The accumulation of cAMP can activate protein kinase A (PKA), making the AC-cAMP-PKA pathway the most important intracellular signaling pathway of IMD. This pathway is involved in the attenuation of vascular calcification (Chen et al., 2020), alleviation of cardiac fibrosis (Zhang et al., 2022), inhibition of atherosclerosis (Dai et al., 2012), reduction of blood pressure, and improvement of cardiac function during hypertension (Yuan et al., 2012), among other effects. IMD can also increase the activity of nitric oxide synthase (NOS) and endothelial NOS (eNOS), leading to elevated nitric oxide (NO) content and enhanced L-arginine uptake in tissues (Chen et al., 2006). This mechanism is involved in the attenuation of hypoxic pulmonary vascular remodeling (Mao et al., 2014). Furthermore, NO can activate guanylate cyclase (GC) and elevate intracellular cGMP levels. Therefore, the L-arginine-NO-cGMP signal pathway also plays a significant role in the biological function of IMD.

Recently, IMD has been reported to mediate various biological functions through multiple pathways (Figure 3). These include the PI3K/Akt and AMPK pathways, which are involved in attenuating aging-associated vascular calcification (Chen et al., 2020). Additionally, IMD has been shown to activate the ERK pathway, promoting cell migration and tube formation in cultured endothelial cells (Smith et al., 2009). In hepatocellular carcinoma, IMD is involved in cell invasion through the ERK1/2-EGR1/DDIT3 signaling pathway (Xiao et al., 2021). Moreover, IMD contributes to decreased atrial fibrosis in rats after myocardial infarction operation through the TGF-β1/Smad3 signaling pathway (Ma et al., 2023). In diabetic conditions, IMD promotes bone generation via the PPARγ/NF-κB signaling pathway (Wang et al., 2021). Furthermore, IMD participates in alleviating pathological cardiac remodeling through the PPARγ-klotho pathway (Zhang et al., 2020).

However, the receptor affinity and activation of downstream signaling pathways remain unclear. In addition, there is a lack of antagonists and analogs that can be utilized to further differentiate the specific pathways of different receptors (Ni et al., 2014). Thus, the development of analogs or specific inhibitors of IMD could be crucial in comprehending the biological functions and molecular mechanisms of IMD.

## 5 Functions of IMD and its family members

### 5.1 Functions of CGRP

The calcitonin gene-related peptide (CGRP) is widely distributed and performs critical biological functions. Calcitonin, CGRP, and amylin are essential regulators of cardiovascular diseases, kidney diseases, sepsis, inflammatory responses, and diabetes (Bell and McDermott, 2008). In a physiological state, the primary role of amylin is to regulate glucose metabolism, while calcitonin regulates calcium metabolism (Wong et al., 2014; Junfeng et al., 2016). CGRP and ADM share similar biological effects, including the regulation of cardiovascular homeostasis and vasodilation (Naot and Cornish, 2008). CGRP is also a neuropeptide that plays a major role in the sensory nervous system (Brain and Grant, 2004).

### 5.2 Functions of ADM

Members of ADM family are all found in fish (Takei et al., 2004). In mammals, only three peptides from ADM family, ADM, ADM2 (IMD), and ADM5 have been identified (Takei et al., 2008). ADM contains 52 amino acids and plays an important role in human tissues and organs in a paracrine manner (Hay et al., 2011). ADM primarily functions through the AM_1_ and AM_2_ receptors (Poyner et al., 2002). ADM is significantly expressed in endothelial cells and may contribute to promoting cardiovascular relaxation (García et al., 2006). ADM is also expressed in various tissues and exerts vasodilatory effects on blood vessels. Voors et al. found that the expression of ADM significantly increased during the progression of chronic heart failure (Voors et al., 2019). The short intermediate peptide pro-AMD_45–92_, generated during ADM hydrolysis, has demonstrated the ability to predict the progression of heart failure in clinical settings (Yuyun et al., 2015).

ADM is also extensively expressed in various types of tumors, and its expression significantly increases in response to hypoxia-inducible factor-1α (HIF-1α) under hypoxic conditions (Albertin et al., 2005). Importantly, high expression of ADM promotes the formation of blood vessels and lymphatic vessels within tumors, which contributes to tumor initiation and progression. ADM has also been implicated in many endocrine-related tumorigenesis, such as renal cell carcinoma (Deville et al., 2009), granulation cell tumor (Deville et al., 2009), hepatocellular carcinoma (Qu et al., 2015), pancreatic cancer cell (Keleg et al., 2007), invasive squamous cell carcinoma (Li et al., 2001), and neuroendocrine tumors (Li et al., 2001).

### 5.3 Functions of IMD

#### 5.3.1 Tumor progression

Recent studies have revealed a strong correlation between IMD and the development of tumors, as well as the prognosis of patients with tumors (Hirose et al., 2008; Ni et al., 2014). In 2008, Morimoto et al. reported for the first time that IMD expression was increased in adrenal tumors (Morimoto et al., 2008). In 2012, Guo et al. discovered that the expression of IMD is relatively low in normal hepatocytes, but it gradually increases with the degree of malignancy in hepatocellular carcinoma (HCC). Subsequent studies have shown that IMD was primarily expressed in malignant hepatic carcinoma cells and in the vicinity of tumor blood vessels, which promotes the proliferation of HCC (Guo et al., 2012). Our team has recently shown that IMD could facilitate metastasis and invasion of HCC through ERK1/2-EGR1/DDIT3 signaling cascade (Xiao et al., 2021). Lu et al. reported that the level of IMD in the peripheral blood of breast cancer patients was significantly higher than that of normal controls. Furthermore, patients who had higher IMD expression before surgery exhibited lower 5-year survival rate and overall survival rate, as well as a higher recurrence rate (Lu et al., 2015). Kong et al. also found that IMD expression was significantly increased in breast cancer samples and facilitated its metastasis by increasing ribosome biogenesis and protein translation via the Src/c-Myc signaling pathway (Kong et al., 2022). Hollander et al. showed that patients with pancreatic adenocarcinoma who had high IMD expression exhibited a shorter median survival time and poorer 5-year survival compared to those with low IMD expression (Hollander et al., 2015).

These studies suggest that IMD and its related peptides are involved in the onset and progression of multiple tumors, potentially playing important roles in cancer cell survival and invasion. Further research is needed to confirm the potential mechanism of IMD and its related peptides in tumorigenesis and development.

#### 5.3.2 Angiogenesis and vascular fusion

The process of angiogenesis is necessary to provide a suitable vascular network for tumor growth and metastasis. The primary characteristics of tumor vessels include an unstable vascular endothelial barrier, increased permeability, and dysregulation of vascular expansion, which facilitate tumor invasion. Thus, controlling tumor-associated angiogenesis is an effective approach to limit tumor progression (Fiedler and Augustin, 2006; Carmeliet and Jain, 2011). The process of blood vessel formation can be divided into two stages: vasculogenesis and angiogenesis (Morin and Tranquillo, 2013; Ibrahim and Richardson, 2017; Vallée et al., 2018). The former refers to the process of establishing a fundamental vascular network from scratch. The latter involves the growth of new blood vessels from the existing primary network, leading to the formation of a functional vascular network (Ribatti et al., 2015).

Emerging evidence demonstrates that IMD, a novel proangiogenic factor, plays an essential role in regulating angiogenesis (Smith et al., 2009). Zhang et al. identified that IMD could promote vascular remodeling and tumor angiogenesis in a tumor vascular model (Zhang et al., 2012). The exogenous IMD_1–47_ peptide was found to enhance blood supply and promote tumor growth (Zhang et al., 2012; Wang et al., 2018). IMD_1–53_ might regulate vessel function homeostasis via upregulating the L-Arg/NOS/NO pathway (Yang et al., 2006). Moreover, studies have demonstrated that IMD promotes endothelial migration and tube formation by increasing the phosphorylation of ERK, AKT, as well as the synthesis of vascular endothelial growth factor (VEGF) and VEGF receptor-2 (VEGFR-2) (Smith et al., 2009). Additionally, IMD could directly activate the phosphorylation of VEGFR-2 through CGRP receptors or AM_1_ receptors, providing an additional mechanism for pro-angiogenesis (Cai et al., 2010).

Regular expression of VEGF results in consistent and stable blood vessel development, whereas overexpression of VEGF leads to an excessive budding of vessels and the formation of irregular blood vessels. Xiao et al. discovered that IMD acted as a proangiogenic factor that can inhibit excessive vascular budding induced by overexpressed VEGF (Xiao et al., 2015). In addition, IMD has the ability to regulate the expression of vascular endothelial-cadherin (VEC) and prevent vascular budding caused by increased endothelial space (Zhang et al., 2012; Xiao et al., 2015). Furthermore, IMD could promote vessel fusion by inducing endothelial cells to enter a “ready-to-anchor” state and regulating VEC activity to achieve a dynamic balance between VEC complex dissociation and reconstitution (Kong et al., 2020).

**FIGURE 4:**
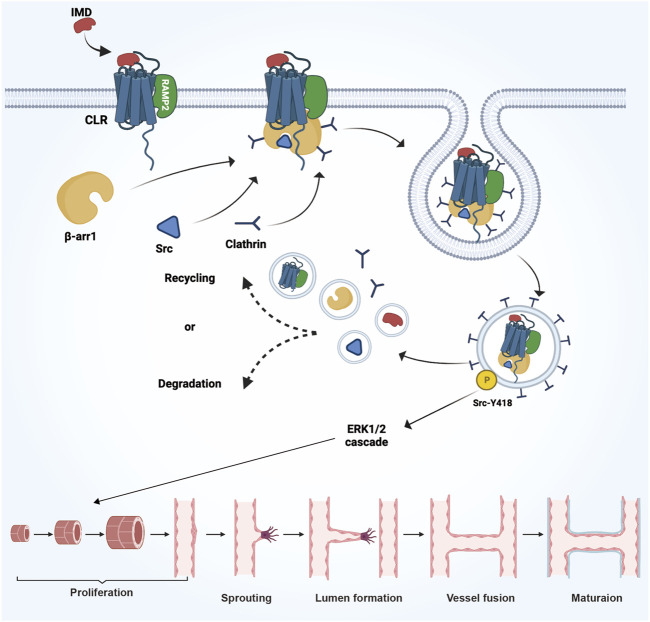
The mechanism and process of IMD promote vascular growth in tumors. IMD regulates the expression of the CLR receptor complex and β-arrestin1/Src, facilitating their translocation into the cytoplasm to activate the downstream ERK1/2 signaling pathway. This activation promotes the expansion of the blood vessel lumen and enhances the maturation of tumor blood vessels.

The expansion of blood vessel lumen is a result of the restored proliferative ability of quiescent endothelial cells, rather than any morphological changes in the endothelial cells themselves. Studies have found that IMD could regulate the expression of CLR receptor complex and β-arrestin1/Src, facilitating their translocation into the cytoplasm to activate the downstream ERK1/2 signaling pathway (Figure 4). This activation could promote the maturation of tumor blood vessels and enhance blood perfusion (Wang et al., 2018).

These mechanisms demonstrate that IMD may play a crucial role in promoting angiogenesis and the fusion of tumor blood vessels, ultimately leading to the establishment of a relatively suitable and functional vascular network for tumor growth.

#### 5.3.3 Hemodynamics

As a potential vascular regulator, IMD plays an important role in regulating systemic hemodynamics and electrolyte balance, as well as protecting against ischemic injury and oxidative stress. IMD is widely expressed in human hypothalamus-pituitary-adrenal axis, as well as in the heart and kidney, suggesting its role in regulating circulation and water-electrolyte metabolism (Takahashi et al., 2006; Takahashi et al., 2011). Administration of IMD into the lateral cerebral ventricle of rats caused significant and long-lasting elevations in mean arterial pressure and heart rate, indicating that IMD had the ability to activate the sympathetic nervous system (Taylor et al., 2005). Evidence suggests that IMD may promote vasodilation, increase renal perfusion, and exhibit direct diuretic effects. Intravenous administration of IMD_1–47_ peptides results in increased blood flow and decreased vascular resistance in various organs, including the heart, lungs, kidneys, liver, stomach, small intestine, and testis (Fujisawa et al., 2007).

Ischemia-reperfusion injury (IRI) is a significant cause of acute kidney injury (AKI) and increases the risk of developing chronic kidney disease (CKD) subsequently. IMD upregulated the mRNA and protein expression levels of VEGF and VEGFR2, while downregulating the expression levels of MMP2, MMP9 and ET-1. Thus, IMD could protect the kidney from ischemia-reperfusion injury by facilitating angiogenesis and reducing the destruction of the perivascular matrix (Wang et al., 2020). Emerging evidence suggests that the protective effects of IMD may be attributed to the regulation of mitochondrial function. Chen et al. found that IMD could mitigate the alterations in mitochondrial membrane induced by oxidative stress in brain endothelial cells (Chen et al., 2006).

#### 5.3.4 Cardiovascular disease

Cardiovascular disease is a leading cause of morbidity and mortality, accounting for 20%–30% of all deaths in the population (Aggarwal et al., 2017; Leong et al., 2017). IMD has a protective effect on the cardiovascular system and cardiometabolic diseases. Much data has demonstrated that changes in the expression levels of IMD and its receptors can affect the development and progression of cardiovascular diseases (Hirose et al., 2008; Yuyun, et al., 2015; Bell et al., 2016). IMD may play both direct and indirect roles in maintaining cardiovascular homeostasis and improving cardiac function. For instance, IMD can directly inhibits myocardial fibrosis by downregulating TGF-β. Additionally, it indirectly regulates metabolic syndrome and reduces the risk factors associated with such diseases (Zhang et al., 2016).

Vascular calcification is considered an independent predictor of cardiovascular morbidity and mortality. IMD is a cardiovascular protective peptide that can suppress vascular calcification in rats by upregulating γ-carboxyglutamic acid (Gla) protein (Cai et al., 2010). IMD infusion significantly reduced atherosclerotic lesion areas and the number of calcified nodules in aortic roots in homocysteine-treated ApoE^−/−^ mice. Mechanistically, IMD alleviated endoplasmic reticulum stress (ERS) activation and decreased the protein levels of ERS markers in vascular smooth muscle cells (VSMCs) and mouse peritoneal macrophages (Ren et al., 2020). Exogenous administration of IMD_1–53_ significantly inhibited the calcium deposition in aortas and the osteogenic trans-differentiation of vascular smooth muscle cells (VSMCs) in vitamin D3 plus nicotine (VDN)-treated old rats. Mechanistically, IMD_1–53_ attenuated aging-associated vascular calcification by upregulating sirt1 via activating PI3K/Akt, AMPK, and cAMP/PKA signaling (Chen et al., 2020). These suggested that IMD is a potential drug target for preventing and treating cardiovascular diseases.

These studies have demonstrated that IMD plays a crucial role in maintaining cardiovascular homeostasis and alleviating vascular diseases. As a result, it has emerged as a potential drug target for the treatment of atherosclerosis, vascular calcification, and hypertension (Ni et al., 2014; Zhang et al., 2018). For instance, the use of recombinant peptide or adeno-associated virus (AAV)-mediated IMD has been investigated as a potential method to mitigate cardiovascular disease (Zhang et al., 2018). However, the clinical application of IMD is still limited due to factors such as unclear mechanisms and insufficient experimental research.

#### 5.3.5 Heart failure

Since the progression of heart failure is irreversible, early diagnosis and treatment are crucial as they make it easier to prevent the disease from worsening and improve the survival rate and quality of life of patients (Coiro et al., 2017; Pei et al., 2018). Studies have suggested that the expression level of IMD could serve as a prognostic indicator for heart failure (Zhang et al., 2022). In a rat model of congestive heart failure induced by left coronary artery ligation, Hirose et al. discovered a significant increase in the expression levels of IMD, CLR, and RAMPs (RAMP1/RAMP2/RAMP3) in cardiac tissues (Hirose et al., 2008). Cabiati et al. found that the expression of IMD significantly increased in the peripheral blood of patients with chronic heart failure (Cabiati et al., 2014). Short peptides derived from pre-pro-IMD_25–56_ and pre-pro-IMD_57-92_ have been detected in serum of human with heart failure (Bell et al., 2016; Arrigo et al., 2017).

Patients with acute heart failure who exhibited high IMD expression in peripheral blood showed stronger myocardial function compared to those with low IMD expression (Gan et al., 2014). Furthermore, in a rat model of angiotensin II (Ang II)-induced cardiac fibrosis, IMD was shown to alleviate cardiac fibrosis by suppressing NLRP3 inflammasome activation and endoplasmic reticulum stress. And this was achieved through inhibition of IRE1α via the cAMP/PKA pathway (Zhang et al., 2022). In rats after myocardial infarction operation, IMD improved atrial fibrosis and reduced the inducibility of atrial fibrillation through the TGF-β1/Smad3 and TGF-β1/Nox4 pathways (Ma et al., 2023). Nevertheless, in clinical practice, it is difficult to accurately determine IMD levels in serum due to its short half-life, the existence of binding proteins and the technical difficulties (Cabiati et al., 2014).

Cardiac remodeling is characterized by cardiac hypertrophy, fibrosis, dysfunction, and eventually progresses to heart failure. Cardiac remodeling was attenuated in mice overexpressing IMD. IMD markedly suppressed the phosphorylation of Ca^2+^/calmodulin-dependent protein kinase II (CaMKII) and the activity of calcineurin to protect against cardiac hypertrophy. This effect was achieved by upregulating klotho both *in vivo* and *in vitro* (Zhang et al., 2020).

#### 5.3.6 Obesity and diabetes

Some studies have demonstrated a tight association between the expression of IMD and type 2 diabetes mellitus (T2DM). There was also a correlation between plasma IMD levels and body mass index (BMI), diastolic blood pressure (DBP), triglycerides, uric acid, blood urea nitrogen, fasting and 2-h postprandial blood glucose, and glycated hemoglobin (HbA1C) (Liu et al., 2022).

IMD is expressed in adipose tissue and is downregulated in diet-induced obese mice and in db/db mice (Zhang et al., 2016). Zhang et al. found that IMD mainly inhibited metabolic syndrome by targeting the adipose tissue (Zhang et al., 2016). An inverse correlation was observed between plasma levels of IMD and BMI. Further studies have revealed that IMD treatment restores the early insulin resistance induced by high-fat diet in adipose tissue, primarily through the inhibition of adipocyte MHCII antigen presentation and CD4^+^ T-cell activation (Zhang et al., 2016; Wang et al., 2021). Systemic administration of synthesized IMD reduced high-fat diet-induced body weight gain, as well as systemic insulin resistance, through activation of the AMP-activated protein kinase (AMPK) pathway and an increase of uncoupling protein 1 (UCP1) expression in adipose tissue (Zhang et al., 2016).

Additionally, IMD could alleviate inflammation in adipose tissue by regulating the number of macrophages present in the tissue (Soultanova et al., 2016). IMD treatment reversed advanced glycation end products-mediated M1 macrophage polarization and impaired bone regeneration in type 1 diabetes mellitus (T1DM), which was partially achieved by the peroxisome proliferator-activated receptor γ (PPARγ)-mediated inhibition of NF-κB signaling (Wang et al., 2021). IMD_1–53_ primarily induces the activation of M2-type macrophages and reverses the elevated M1/M2 macrophage ratio by inhibiting AMPK activity. This mechanism contributes to the reduction of inflammation in adipose tissue (Pang et al., 2016). Furthermore, during obesity, adipocytes mainly express MHC-II and function as antigen-presenting cells, initiating adaptive immune responses. Since macrophages express receptors for IMD, it could downregulate MHC-II expression in adipocytes through a cAMP-BlimP1-dependent manner (Deng et al., 2013). Based on a mouse model of atherosclerosis in diabetes, IMD attenuates macrophage phagocytosis through regulation of the long noncoding RNA Dnm3os/miR-27b-3p/SLAMF7 signaling pathway (Su et al., 2021).

#### 5.3.7 Sepsis

Sepsis is a systemic inflammatory response syndrome and the most common critical illness in the intensive care unit (ICU), with the highest mortality rate (Angus and van der Poll, 2013; Minasyan, 2019). Despite recent advances in sepsis treatment, the mortality rate still ranges from 30% to 60% (Laroye et al., 2017; Peterzan et al., 2017). Zhang et al. conducted a retrospective analysis of clinical data from elderly patients with sepsis and found significant increases in serum levels of IMD, C-reactive protein (CRP), and procalcitonin (PCT) (Zhang et al., 2017). These findings suggest that serum IMD may serve as an indicator of the severity and prognosis of sepsis in elderly patients.

Clinical findings indicate that IMD has a protective effect in the development of sepsis and can serve as a specific marker for clinical diagnosis (Xiao et al., 2018). Vascular leakage and cytokine storms are two typical pathological characteristics of sepsis (Aird, 2003; Deutschman and Tracey, 2014; Armstrong et al., 2017). Xiao et al. discovered that IMD could provide protection against organ damage caused by vascular leakage and inflammatory response. Subsequent studies have shown that IMD could reconstruct the endothelial barrier by regulating Rab11 to promote VEC transport, which can repair the endothelial space and alleviate vascular leakage (Xiao et al., 2018).

Sepsis causes myocardial damage in approximately 40% of patients (Hato et al., 2019). Myocardial dysfunction is responsible for the death of approximately 70%–90% of sepsis patients (Hoover et al., 2015). Studies have shown that IMD can relax blood vessels, reduce blood pressure, and provide protection against myocardial ischemia (Dai et al., 2012; Mao et al., 2014). Zhu et al. discovered, in a rat model of sepsis induced by CLP, that administering exogenous IMD could increase oxygen transport, improve tissue perfusion, and enhance myocardial contractility and function (Zhu et al., 2016). Moreover, the administration of IMD peptides prior to surgery has been shown to significantly improve survival rates (Zhu et al., 2016). Interleukin-1β (IL-1β) is an inflammatory factor that plays a crucial role in the development of tissue and organ damage during sepsis. Studies have shown that IMD could reduce the expression of major inflammatory factors in sepsis by regulating the NLRP3/Caspase-1/IL-1β pathway, thereby exerting a protective effect against sepsis-induced cardiac dysfunction (Johnson et al., 2018).

Taken together, the expression level of IMD is closely associated with the development of sepsis. Thus, the levels of IMD or pre-pro-IMD peptide in plasma can serve as diagnostic markers or potential drug targets for septic syndromes. To translate the research on IMD into clinical applications, it is necessary to further elucidate the role of IMD in sepsis.

## 6 Conclusion

**FIGURE 5:**
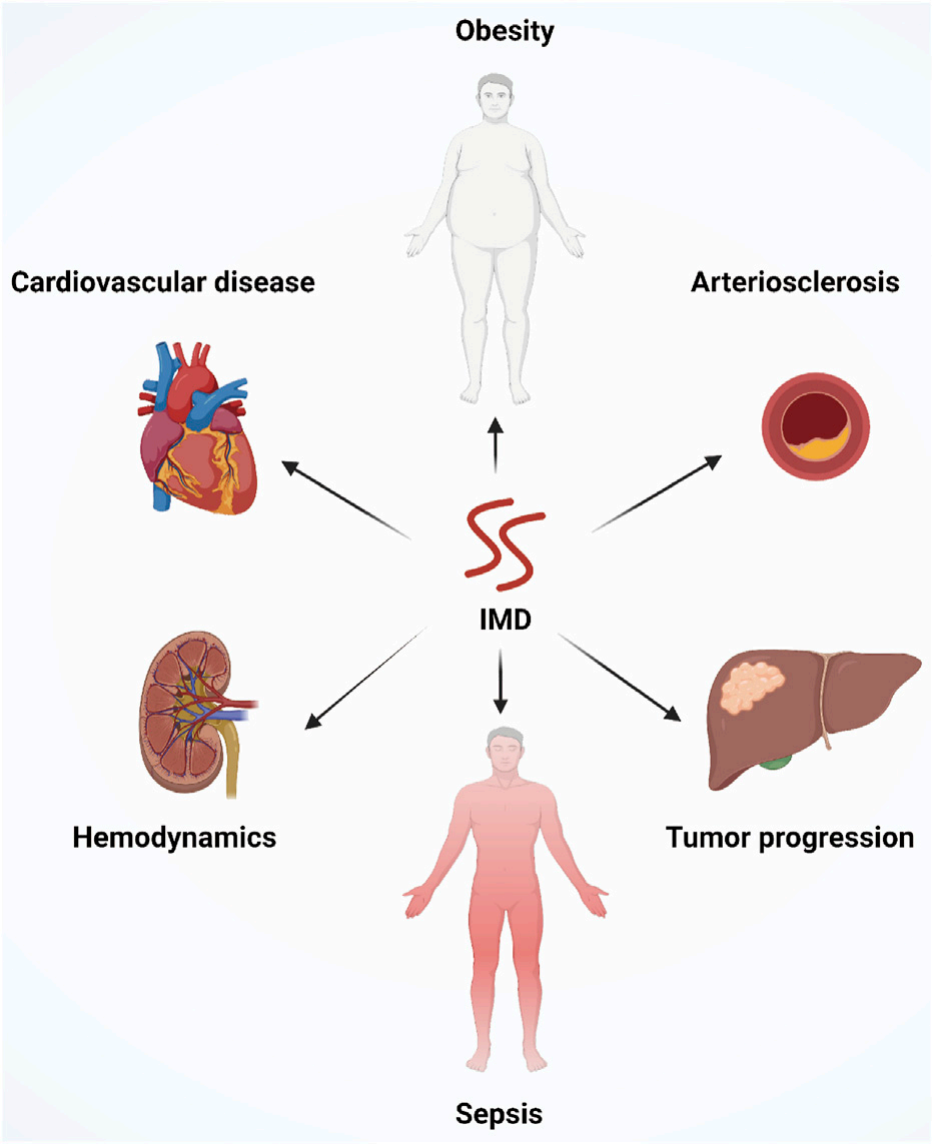
An overview of the multiple physiological functions of IMD, including improving cardiovascular diseases, alleviating atherosclerosis, regulating hemodynamics, improving obesity and metabolic syndrome, controlling systemic inflammation, promoting angiogenesis and tumor progression.

Altogether, the crucial role of IMD in regulating vascular development, cardiovascular disease, cancer, and inflammatory diseases is fundamental for its application in clinical practice (Figure 5). In-depth studies are needed to explore the signal transduction pathways of IMD and the specific roles of different active segments of IMD in regulating gene expression, biological effect, receptor binding, receptor signal transduction, as well as the genesis and development of diseases.

## References

[B1] Aggarwal M. Aggarwal B. Rao J. (2017). Integrative medicine for cardiovascular disease and prevention. Med. Clin. North Am. 101 (5), 895–923. 10.1016/j.mcna.2017.04.007 28802470

[B2] Aird W. C. (2003). The role of the endothelium in severe sepsis and multiple organ dysfunction syndrome. Blood 101 (10), 3765–3777. 10.1182/blood-2002-06-1887 12543869

[B3] Albertin G. Carraro G. Petrelli L. Guidolin D. Neri G. Nussdorfer G. G. (2005). Endothelin-1 and adrenomedullin enhance the growth of human adrenocortical carcinoma-derived SW-13 cell line by stimulating proliferation and inhibiting apoptosis. Int. J. Mol. Med. 15 (3), 469–474. 10.3892/ijmm.15.3.469 15702240

[B4] Alexander S. P. Davenport A. P. Kelly E. Marrion N. Peters J. A. Benson H. E. (2015). The concise guide to PHARMACOLOGY 2015/16: g protein-coupled receptors. Br. J. Pharmacol. 172 (24), 5744–5869. 10.1111/bph.13348 26650439 PMC4718210

[B5] Angus D. C. van der Poll T. (2013). Severe sepsis and septic shock. N. Engl. J. Med. 369 (9), 2063–2851. 10.1056/NEJMc1312359 24256390

[B6] Armstrong B. A. Betzold R. D. May A. K. (2017). Sepsis and septic shock strategies. Surg. Clin. North Am. 97 (6), 1339–1379. 10.1016/j.suc.2017.07.003 29132513

[B7] Arrigo M. Truong Q. A. Szymonifka J. Rivas-Lasarte M. Tolppanen H. Sadoune M. (2017). Mid-regional pro-atrial natriuretic peptide to predict clinical course in heart failure patients undergoing cardiac resynchronization therapy. Europace 19 (11), 1848–1854. 10.1093/europace/euw305 28096288 PMC5834135

[B8] Bailey R. J. Hay D. L. (2006). Pharmacology of the human CGRP1 receptor in cos 7 cells. Peptides 27 (6), 1367–1375. 10.1016/j.peptides.2005.11.014 16375989

[B9] Bell D. Campbell M. Ferguson M. Sayers L. Donaghy L. O'Regan A. (2012). AM₁-receptor-dependent protection by intermedin of human vascular and cardiac non-vascular cells from ischaemia-reperfusion injury. J. Physiol. 590 (5), 1181–1197. 10.1113/jphysiol.2011.221895 22183724 PMC3381824

[B10] Bell D. Campbell M. McAleer S. F. Ferguson M. Donaghy L. Harbinson M. T. (2016). Endothelium-derived intermedin/adrenomedullin-2 protects human ventricular cardiomyocytes from ischaemia-reoxygenation injury predominantly via the AM- receptor. Peptides 76, 1–13. 10.1016/j.peptides.2015.12.005 26743504

[B11] Bell D. Gordon B. J. Lavery A. Megaw K. Kinney M. O. Harbinson M. T. (2016). Plasma levels of intermedin (adrenomedullin-2) in healthy human volunteers and patients with heart failure. Peptides 76, 19–29. 10.1016/j.peptides.2015.12.003 26767798

[B12] Bell D. McDermott B. J. (2008). Intermedin (adrenomedullin-2): a novel counter-regulatory peptide in the cardiovascular and renal systems. Br. J. Pharmacol. 153, S247–S262. 10.1038/sj.bjp.0707494 17965749 PMC2268039

[B13] Booe J. M. Warner M. L. Roehrkasse A. M. Hay D. L. Pioszak A. A. (2018). Probing the mechanism of receptor activity-modifying protein modulation of GPCR ligand selectivity through rational design of potent adrenomedullin and calcitonin gene-related peptide antagonists. Mol. Pharmacol. 93 (4), 355–367. 10.1124/mol.117.110916 29363552 PMC5832325

[B14] Brain S. D. Grant A. D. (2004). Vascular actions of calcitonin gene-related peptide and adrenomedullin. Physiol. Rev. 84 (3), 903–934. 10.1152/physrev.00037.2003 15269340

[B15] Cabiati M. Sabatino L. Svezia B. Caruso R. Verde A. Caselli C. (2014). Adrenomedullin and intermedin gene transcription is increased in leukocytes of patients with chronic heart failure at different stages of the disease. Peptides 55, 13–16. 10.1016/j.peptides.2014.01.028 24531032

[B16] Cai Y. Xu M. J. Teng X. Zhou Y. B. Chen L. Zhu Y. (2010). Intermedin inhibits vascular calcification by increasing the level of matrix gamma-carboxyglutamic acid protein. Cardiovasc Res. 85 (4), 864–873. 10.1093/cvr/cvp366 19910445

[B17] Carmeliet P. Jain R. K. (2011). Molecular mechanisms and clinical applications of angiogenesis. Nature 473 (7347), 298–307. 10.1038/nature10144 21593862 PMC4049445

[B18] Chang C. L. Roh J. Hsu S. Y. (2004). Intermedin, a novel calcitonin family peptide that exists in teleosts as well as in mammals: a comparison with other calcitonin/intermedin family peptides in vertebrates. Peptides 25 (10), 1633–1642. 10.1016/j.peptides.2004.05.021 15476930

[B19] Chen L. Kis B. Hashimoto H. Busija D. W. Takei Y. Yamashita H. (2006). Adrenomedullin 2 protects rat cerebral endothelial cells from oxidative damage *in vitro* . Brain Res. 1086 (1), 42–49. 10.1016/j.brainres.2006.02.128 16616051

[B20] Chen Y. Zhang L. S. Ren J. L. Zhang Y. R. Wu N. Jia M. Z. (2020). Intermedin(1-53) attenuates aging-associated vascular calcification in rats by upregulating sirtuin 1. Aging (Albany NY) 12 (7), 5651–5674. 10.18632/aging.102934 32229709 PMC7185112

[B21] Coiro S. Girerd N. Rossignol P. Ferreira J. P. Maggioni A. Pitt B. (2017). Association of beta-blocker treatment with mortality following myocardial infarction in patients with chronic obstructive pulmonary disease and heart failure or left ventricular dysfunction: a propensity matched-cohort analysis from the high-risk myocardial infarction database initiative. Eur. J. Heart Fail 19 (2), 271–279. 10.1002/ejhf.647 27774703

[B22] Dai X. Y. Cai Y. Mao D. D. Qi Y. F. Tang C. Xu Q. (2012). Increased stability of phosphatase and tensin homolog by intermedin leading to scavenger receptor A inhibition of macrophages reduces atherosclerosis in apolipoprotein E-deficient mice. J. Mol. Cell Cardiol. 53 (4), 509–520. 10.1016/j.yjmcc.2012.07.006 22841663

[B23] Deng T. Lyon C. J. Minze L. J. Lin J. Zou J. Liu J. Z. (2013). Class II major histocompatibility complex plays an essential role in obesity-induced adipose inflammation. Cell Metab. 17 (3), 411–422. 10.1016/j.cmet.2013.02.009 23473035 PMC3619392

[B24] Deutschman C. S. Tracey K. J. (2014). Sepsis: current dogma and new perspectives. Immunity 40 (4), 463–475. 10.1016/j.immuni.2014.04.001 24745331

[B25] Deville J. L. Bartoli C. Berenguer C. Fernandez-Sauze S. Kaafarani I. Delfino C. (2009). Expression and role of adrenomedullin in renal tumors and value of its mRNA levels as prognostic factor in clear-cell renal carcinoma. Int. J. Cancer 125 (10), 2307–2315. 10.1002/ijc.24568 19610056

[B26] Fiedler U. Augustin H. G. (2006). Angiopoietins: a link between angiogenesis and inflammation. Trends Immunol. 27 (12), 552–558. 10.1016/j.it.2006.10.004 17045842

[B27] Fujisawa Y. Nagai Y. Miyatake A. Miura K. Nishiyama A. Kimura S. (2007). Effects of adrenomedullin 2 on regional hemodynamics in conscious rats. Eur. J. Pharmacol. 558 (1-3), 128–132. 10.1016/j.ejphar.2006.11.043 17204266

[B28] Gan X. B. Sun H. J. Chen D. Zhang L. L. Zhou H. Chen L. Y. (2014). Intermedin in the paraventricular nucleus attenuates cardiac sympathetic afferent reflex in chronic heart failure rats. PLoS One 9 (4), e94234. 10.1371/journal.pone.0094234 24709972 PMC3978024

[B29] García M. A. Martín-Santamaría S. de Pascual-Teresa B. Ramos A. Julián M. Martínez A. (2006). Adrenomedullin: a new and promising target for drug discovery. Expert Opin. Ther. Targets 10 (2), 303–317. 10.1517/14728222.10.2.303 16548778

[B30] García-Ponce A. Chánez Paredes S. Castro Ochoa K. F. Schnoor M. (2016). Regulation of endothelial and epithelial barrier functions by peptide hormones of the adrenomedullin family. Tissue Barriers 4 (4), e1228439. 10.1080/21688370.2016.1228439 28123925 PMC5214765

[B31] Guo X. Schmitz J. C. Kenney B. C. Uchio E. M. Kulkarni S. Cha C. H. (2012). Intermedin is overexpressed in hepatocellular carcinoma and regulates cell proliferation and survival. Cancer Sci. 103 (8), 1474–1480. 10.1111/j.1349-7006.2012.02341.x 22625651 PMC7659195

[B32] Hato T. Maier B. Syed F. Myslinski J. Zollman A. Plotkin Z. (2019). Bacterial sepsis triggers an antiviral response that causes translation shutdown. J. Clin. Invest. 129 (1), 296–309. 10.1172/JCI123284 30507610 PMC6307966

[B33] Hay D. L. Pioszak A. A. (2016). Receptor activity-modifying proteins (RAMPs): new insights and roles. Annu. Rev. Pharmacol. Toxicol. 56, 469–487. 10.1146/annurev-pharmtox-010715-103120 26514202 PMC5559101

[B34] Hay D. L. Walker C. S. Poyner D. R. (2011). Adrenomedullin and calcitonin gene-related peptide receptors in endocrine-related cancers: opportunities and challenges. Endocr. Relat. Cancer 18 (1), C1–C14. 10.1677/ERC-10-0244 21051558

[B35] Hirose T. Totsune K. Mori N. Morimoto R. Hashimoto M. Nakashige Y. (2008). Increased expression of adrenomedullin 2/intermedin in rat hearts with congestive heart failure. Eur. J. Heart Fail 10 (9), 840–849. 10.1016/j.ejheart.2008.06.020 18692436

[B36] Hollander L. L. Guo X. Salem R. R. Cha C. H. (2015). The novel tumor angiogenic factor, adrenomedullin-2, predicts survival in pancreatic adenocarcinoma. J. Surg. Res. 197 (2), 219–224. 10.1016/j.jss.2014.11.002 25982376

[B37] Hoover D. B. Ozment T. R. Wondergem R. Li C. Williams D. L. (2015). Impaired heart rate regulation and depression of cardiac chronotropic and dromotropic function in polymicrobial sepsis. Shock 43 (2), 185–191. 10.1097/SHK.0000000000000272 25271380 PMC4297223

[B38] Ibrahim M. Richardson M. K. (2017). Beyond organoids: *in vitro* vasculogenesis and angiogenesis using cells from mammals and zebrafish. Reprod. Toxicol. 73, 292–311. 10.1016/j.reprotox.2017.07.002 28697965

[B39] Johnson M. O. Wolf M. M. Madden M. Z. Andrejeva G. Sugiura A. Contreras D. C. (2018). Distinct regulation of Th17 and Th1 cell differentiation by glutaminase-dependent metabolism. Cell 175 (7), 1780–1795. 10.1016/j.cell.2018.10.001 30392958 PMC6361668

[B40] Junfeng G. Huiyu Z. Gang Z. Yang A. Yang Y. Fei W. (2016). Protective effect of calcitonin gene-related peptide against oxidative damage in MC3T3-E1 osteoblasts. Hua Xi Kou Qiang Yi Xue Za Zhi 34 (6), 584–588. 10.7518/hxkq.2016.06.007 28318158 PMC7030866

[B41] Keleg S. Kayed H. Jiang X. Penzel R. Giese T. Büchler M. W. (2007). Adrenomedullin is induced by hypoxia and enhances pancreatic cancer cell invasion. Int. J. Cancer 121 (1), 21–32. 10.1002/ijc.22596 17290391

[B42] Kong L. Xiao F. Wang L. Li M. Wang D. Feng Z. (2020). Intermedin promotes vessel fusion by inducing VE-cadherin accumulation at potential fusion sites and to achieve a dynamic balance between VE-cadherin-complex dissociation/reconstitution. MedComm 1(1), 84–102. 10.1002/mco2.9 34766111 PMC8489673

[B43] Kong L. Xiong Y. Wang D. Huang L. Li M. Feng Z. (2022). Intermedin (adrenomedullin 2) promotes breast cancer metastasis via Src/c-Myc-mediated ribosome production and protein translation. Breast Cancer Res. Treat. 195 (2), 91–103. 10.1007/s10549-022-06687-0 35896852

[B44] Laroye C. Gibot S. Reppel L. Bensoussan D. (2017). Concise review: mesenchymal stromal/stem cells: a new treatment for sepsis and septic shock? Stem Cells 35 (12), 2331–2339. 10.1002/stem.2695 28856759

[B45] Leong D. P. Joseph P. G. McKee M. Anand S. S. Teo K. K. Schwalm J. D. (2017). Reducing the global burden of cardiovascular disease, Part 2: prevention and treatment of cardiovascular disease. Circ. Res. 121 (6), 695–710. 10.1161/CIRCRESAHA.117.311849 28860319

[B46] Li Z. Takeuchi S. Otani T. Maruo T. (2001). Implications of adrenomedullin expression in the invasion of squamous cell carcinoma of the uterine cervix. Int. J. Clin. Oncol. 6 (6), 263–270. 10.1007/s10147-001-8026-8 11828944

[B47] Liu F. Duan J. T. Teng X. Peng D. Q. (2022). The increased plasma levels of intermedin in patients with type 2 diabetes mellitus. Acta Endocrinol. (Buchar) 18 (3), 271–277. 10.4183/aeb.2022.271 36699172 PMC9867815

[B48] Lu Y. M. Zhong J. B. Wang H. Y. Yu X. F. Li Z. Q. (2015). "The prognostic value of intermedin in patients with breast cancer." Dis. Markers 2015, 862158. 10.1155/2015/862158 25694747 PMC4324930

[B49] Ma S. Yan F. Hou Y. (2023). Intermedin 1-53 ameliorates atrial fibrosis and reduces inducibility of atrial fibrillation via TGF-β1/pSmad3 and Nox4 pathway in a rat model of heart failure. J. Clin. Med. 12 (4), 1537. 10.3390/jcm12041537 36836072 PMC9959393

[B50] Mao S. Z. Fan X. F. Xue F. Chen R. Chen X. Y. Yuan G. S. (2014). Intermedin modulates hypoxic pulmonary vascular remodeling by inhibiting pulmonary artery smooth muscle cell proliferation. Pulm. Pharmacol. Ther. 27 (1), 1–9. 10.1016/j.pupt.2013.06.004 23796770

[B51] Minasyan H. (2019). Sepsis: mechanisms of bacterial injury to the patient. Scand. J. Trauma Resusc. Emerg. Med. 27 (1), 19. 10.1186/s13049-019-0596-4 30764843 PMC6376788

[B52] Morimoto R. Satoh F. Murakami O. Hirose T. Totsune K. Imai Y. (2008). Expression of adrenomedullin 2/intermedin in human adrenal tumors and attached non-neoplastic adrenal tissues. J. Endocrinol. 198 (1), 175–183. 10.1677/JOE-08-0103 18460550

[B53] Morin K. T. Tranquillo R. T. (2013). *In vitro* models of angiogenesis and vasculogenesis in fibrin gel. Exp. Cell Res. 319 (16), 2409–2417. 10.1016/j.yexcr.2013.06.006 23800466 PMC3919069

[B54] Naot D. Cornish J. (2008). The role of peptides and receptors of the calcitonin family in the regulation of bone metabolism. Bone 43 (5), 813–818. 10.1016/j.bone.2008.07.003 18687416

[B55] Ni X. Zhang J. Tang C. Qi Y. (2014). Intermedin/adrenomedullin2: an autocrine/paracrine factor in vascular homeostasis and disease. Sci. China Life Sci. 57 (8), 781–789. 10.1007/s11427-014-4701-7 25104450

[B56] Ogoshi M. Inoue K. Takei Y. (2003). Identification of a novel adrenomedullin gene family in teleost fish. Biochem. Biophys. Res. Commun. 311 (4), 1072–1077. 10.1016/j.bbrc.2003.10.111 14623291

[B57] Owji A. A. Chabot J.-G. Dumont Y. Quirion R. (2008). Adrenomedullin-2/Intermedin induces cAMP accumulation in dissociated rat spinal cord cells: evidence for the existence of a distinct class of sites of action. J. Mol. Neurosci. 35 (3), 355–361. 10.1007/s12031-008-9062-x 18418734

[B58] Pang Y. Li Y. Lv Y. Sun L. Zhang S. Li Y. (2016). Intermedin restores hyperhomocysteinemia-induced macrophage polarization and improves insulin resistance in mice. J. Biol. Chem. 291 (23), 12336–12345. 10.1074/jbc.M115.702654 27080257 PMC4933280

[B59] Pei H. Wang W. Zhao D. Wang L. Su G. H. Zhao Z. (2018). The use of a novel non-steroidal mineralocorticoid receptor antagonist finerenone for the treatment of chronic heart failure: a systematic review and meta-analysis. Med. Baltim. 97 (16), e0254. 10.1097/MD.0000000000010254 PMC5916685 29668577

[B60] Peterzan M. A. Lygate C. A. Neubauer S. Rider O. J. (2017). Metabolic remodeling in hypertrophied and failing myocardium: a review. Am. J. Physiol. Heart Circ. Physiol. 313 (3), H597–h616. 10.1152/ajpheart.00731.2016 28646030

[B61] Poyner D. R. Sexton P. M. Marshall I. Smith D. M. Quirion R. Born W. (2002). International Union of Pharmacology. XXXII. The mammalian calcitonin gene-related peptides, adrenomedullin, amylin, and calcitonin receptors. Pharmacol. Rev. 54 (2), 233–246. 10.1124/pr.54.2.233 12037140

[B62] Qu Z. Jiang Y. Xu M. Lu M. Z. Zhou B. Ding Y. (2015). Correlation of adrenomedullin with the erythropoietin receptor and microvessel density in hepatocellular carcinoma. Arch. Med. Sci. 11 (5), 978–981. 10.5114/aoms.2015.54852 26528339 PMC4624742

[B63] Ren J. L. Hou Y. L. Ni X. Q. Zhu Q. Chen Y. Zhang L. S. (2020). Intermedin(1-53) ameliorates homocysteine-promoted atherosclerotic calcification by inhibiting endoplasmic reticulum stress. J. Cardiovasc Pharmacol. Ther. 25 (3), 251–264. 10.1177/1074248419885633 31698947

[B64] Ribatti D. Nico B. Crivellato E. (2015). The development of the vascular system: a historical overview. Methods Mol. Biol. 1214, 1–14. 10.1007/978-1-4939-1462-3_1 25468595

[B65] Roehrkasse A. M. Booe J. M. Lee S. M. Warner M. L. Pioszak A. A. (2018). Structure-function analyses reveal a triple β-turn receptor-bound conformation of adrenomedullin 2/intermedin and enable peptide antagonist design. J. Biol. Chem. 293 (41), 15840–15854. 10.1074/jbc.RA118.005062 30139742 PMC6187615

[B66] Roh J. Chang C. L. Bhalla A. Klein C. Hsu S. Y. T. (2004). Intermedin is a calcitonin/calcitonin gene-related peptide family peptide acting through the calcitonin receptor-like receptor/receptor activity-modifying protein receptor complexes. J. Biol. Chem. 279 (8), 7264–7274. 10.1074/jbc.M305332200 14615490

[B67] Smith R. S. Jr. Gao L. Bledsoe G. Chao L. Chao J. (2009). Intermedin is a new angiogenic growth factor. Am. J. Physiol. Heart Circ. Physiol. 297 (3), H1040–H1047. 10.1152/ajpheart.00404.2009 19592612 PMC2755985

[B68] Soultanova A. Mikulski Z. Pfeil U. Grau V. Kummer W. (2016). Calcitonin peptide family members are differentially regulated by LPS and inhibit functions of rat alveolar NR8383 macrophages. PLoS One 11 (10), e0163483. 10.1371/journal.pone.0163483 27737007 PMC5063294

[B69] Su Y. Guan P. Li D. Hang Y. Ye X. Han L. (2021). Intermedin attenuates macrophage phagocytosis via regulation of the long noncoding RNA Dnm3os/miR-27b-3p/SLAMF7 axis in a mouse model of atherosclerosis in diabetes. Biochem. Biophys. Res. Commun. 583, 35–42. 10.1016/j.bbrc.2021.10.038 34717123

[B70] Takahashi K. Kikuchi K. Maruyama Y. Urabe T. Nakajima K. Sasano H. (2006). Immunocytochemical localization of adrenomedullin 2/intermedin-like immunoreactivity in human hypothalamus, heart and kidney. Peptides 27 (6), 1383–1389. 10.1016/j.peptides.2005.11.004 16359754

[B71] Takahashi K. Morimoto R. Hirose T. Satoh F. Totsune K. (2011). Adrenomedullin 2/intermedin in the hypothalamo-pituitary-adrenal axis. J. Mol. Neurosci. 43 (2), 182–192. 10.1007/s12031-010-9413-2 20596793

[B72] Takei Y. Hashimoto H. Inoue K. Osaki T. Yoshizawa-Kumagaye K. Tsunemi M. (2008). Central and peripheral cardiovascular actions of adrenomedullin 5, a novel member of the calcitonin gene-related peptide family, in mammals. J. Endocrinol. 197 (2), 391–400. 10.1677/JOE-07-0541 18434369

[B73] Takei Y. Inoue K. Ogoshi M. Kawahara T. Bannai H. Miyano S. (2004). Identification of novel adrenomedullin in mammals: a potent cardiovascular and renal regulator. FEBS Lett. 556 (1-3), 53–58. 10.1016/s0014-5793(03)01368-1 14706825

[B74] Taylor M. M. Bagley S. L. Samson W. K. (2005). Intermedin/adrenomedullin-2 acts within central nervous system to elevate blood pressure and inhibit food and water intake. Am. J. Physiol. Regul. Integr. Comp. Physiol. 288 (4), R919–R927. 10.1152/ajpregu.00744.2004 15576658

[B75] Vallée A. Guillevin R. Vallée J. N. (2018). Vasculogenesis and angiogenesis initiation under normoxic conditions through Wnt/β-catenin pathway in gliomas. Rev. Neurosci. 29 (1), 71–91. 10.1515/revneuro-2017-0032 28822229

[B76] Voors A. A. Kremer D. Geven C. Ter Maaten J. M. Struck J. Bergmann A. (2019). Adrenomedullin in heart failure: pathophysiology and therapeutic application. Eur. J. Heart Fail 21 (2), 163–171. 10.1002/ejhf.1366 30592365 PMC6607488

[B77] Wang F. Kong L. Wang W. Shi L. Wang M. Chai Y. (2021). Adrenomedullin 2 improves bone regeneration in type 1 diabetic rats by restoring imbalanced macrophage polarization and impaired osteogenesis. Stem Cell Res. Ther. 12 (1), 288. 10.1186/s13287-021-02368-9 33985585 PMC8117361

[B78] Wang L. J. Xiao F. Kong L. M. Wang D. N. Li H. Y. Wei Y. G. (2018). Intermedin enlarges the vascular lumen by inducing the quiescent endothelial cell proliferation. Arterioscler. Thromb. Vasc. Biol. 38 (2), 398–413. 10.1161/ATVBAHA.117.310317 29242270

[B79] Wang Y. Mi Y. Tian J. Qiao X. Su X. Kang J. (2020). Intermedin alleviates renal ischemia-reperfusion injury and enhances neovascularization in wistar rats. Drug Des. Devel Ther. 14, 4825–4834. 10.2147/DDDT.S253019 PMC7666991 33204068

[B80] Weston C. Lu J. Li N. Barkan K. Richards G. O. Roberts D. J. (2015). Modulation of glucagon receptor pharmacology by receptor activity-modifying protein-2 (RAMP2). J. Biol. Chem. 290 (38), 23009–23022. 10.1074/jbc.M114.624601 26198634 PMC4645630

[B81] Weston C. Winfield I. Harris M. Hodgson R. Shah A. Dowell S. J. (2016). Receptor activity-modifying protein-directed G protein signaling specificity for the calcitonin gene-related peptide family of receptors. J. Biol. Chem. 291 (42), 21925–21944. 10.1074/jbc.M116.751362 27566546 PMC5063977

[B82] Wong H. K. Tang F. Cheung T. T. Cheung B. M. (2014). Adrenomedullin and diabetes. World J. Diabetes 5 (3), 364–371. 10.4239/wjd.v5.i3.364 24936257 PMC4058740

[B83] Xiao F. Li H. Feng Z. Huang L. Kong L. Li M. (2021). Intermedin facilitates hepatocellular carcinoma cell survival and invasion via ERK1/2-EGR1/DDIT3 signaling cascade. Sci. Rep. 11 (1), 488. 10.1038/s41598-020-80066-x 33436794 PMC7803743

[B84] Xiao F. Wang D. Kong L. Li M. Feng Z. Shuai B. (2018). Intermedin protects against sepsis by concurrently re-establishing the endothelial barrier and alleviating inflammatory responses. Nat. Commun. 9 (1), 2644. 10.1038/s41467-018-05062-2 29980671 PMC6035189

[B85] Xiao F. Wang L. J. Zhao H. Tan C. Wang D. N. Zhang H. (2015). Intermedin restricts vessel sprouting by inhibiting the loosening of endothelial junction. Biochem. Biophys. Res. Commun. 458 (1), 174–179. 10.1016/j.bbrc.2015.01.090 25637664

[B86] Yang J. H. Pan C. S. Jia Y. X. Zhang J. Zhao J. Pang Y. Z. (2006). Intermedin1-53 activates L-arginine/nitric oxide synthase/nitric oxide pathway in rat aortas. Biochem. Biophys. Res. Commun. 341 (2), 567–572. 10.1016/j.bbrc.2006.01.010 16434024

[B87] Yuan Y. Wang X. Zeng Q. Wu H. M. Qi Y. F. Tang C. S. (2012). Effects of continuous intermedin infusion on blood pressure and hemodynamic function in spontaneously hypertensive rats. J. Geriatr. Cardiol. 9 (1), 17–27. 10.3724/SP.J.1263.2012.00017 22783319 PMC3390097

[B88] Yuyun M. F. Narayan H. K. Ng L. L. (2015). Prognostic significance of adrenomedullin in patients with heart failure and with myocardial infarction. Am. J. Cardiol. 115 (7), 986–991. 10.1016/j.amjcard.2015.01.027 25682438

[B89] Zhang H. Zhang S. Y. Jiang C. Li Y. Xu G. Xu M. J. (2016). Intermedin/adrenomedullin 2 polypeptide promotes adipose tissue browning and reduces high-fat diet-induced obesity and insulin resistance in mice. Int. J. Obes. (Lond) 40 (5), 852–860. 10.1038/ijo.2016.2 26786353

[B90] Zhang L. S. Liu Y. Chen Y. Ren J. L. Zhang Y. R. Yu Y. R. (2020). Intermedin alleviates pathological cardiac remodeling by upregulating klotho. Pharmacol. Res. 159, 104926. 10.1016/j.phrs.2020.104926 32502636

[B91] Zhang L. S. Zhang J. S. Hou Y. L. Lu W. W. Ni X. Q. Lin F. (2022). Intermedin(1-53) inhibits NLRP3 inflammasome activation by targeting IRE1α in cardiac fibrosis. Inflammation 45 (4), 1568–1584. 10.1007/s10753-022-01642-z 35175495

[B92] Zhang S. Y. Lv Y. Zhang H. Gao S. Wang T. Feng J. (2016). Adrenomedullin 2 improves early obesity-induced adipose insulin resistance by inhibiting the class II MHC in adipocytes. Diabetes 65 (8), 2342–2355. 10.2337/db15-1626 27207558

[B93] Zhang S. Y. Xu M. J. Wang X. (2018). Adrenomedullin 2/intermedin: a putative drug candidate for treatment of cardiometabolic diseases. Br. J. Pharmacol. 175 (8), 1230–1240. 10.1111/bph.13814 28407200 PMC5867024

[B94] Zhang W. Wang L. J. Xiao F. Wei Y. Ke W. Xin H. B. (2012). Intermedin: a novel regulator for vascular remodeling and tumor vessel normalization by regulating vascular endothelial-cadherin and extracellular signal-regulated kinase. Arterioscler. Thromb. Vasc. Biol. 32 (11), 2721–2732. 10.1161/ATVBAHA.112.300185 22922959

[B95] Zhang Y. Zhu F. Du W. He Y. Guo L. Yan Z. (2017). Evaluation function of intermedin on prognosis of elderly patients with sepsis. Zhonghua Wei Zhong Bing Ji Jiu Yi Xue 29 (8), 679–683. 10.3760/cma.j.issn.2095-4352.2017.08.002 28795663

[B96] Zhu Y. Wu H. Wu Y. Zhang J. Peng X. Zang J. (2016). Beneficial effect of intermedin 1-53 in septic shock rats: contributions of rho kinase and BKCA pathway-mediated improvement in cardiac function. Shock 46 (5), 557–565. 10.1097/SHK.0000000000000639 27355401

